# Ultrasound-based assessment of spinal muscle thickness and elasticity in patients with idiopathic scoliosis

**DOI:** 10.1186/s13089-025-00455-z

**Published:** 2025-10-14

**Authors:** Huangwei Lei, Lin Meng, Yifan Sun, Yanyun Gou

**Affiliations:** 1 Fujian University of Traditional Chinese Medicine, No.1 Qiuyang road, Minhou Shangjie, Fuzhou, 350122 Fujian China; 2 Hangzhou First Peoples’ Hospital Xiasha Campus, Hangzhou Rehabilitation Hospital, Hangzhou, 310018 Zhejiang China

**Keywords:** Idiopathic scoliosis, Ultrasound, Muscle thickness, Muscle elasticity

## Abstract

**Purpose:**

Utilizing ultrasonic imaging technology, this study assessed and compared the thickness and elasticity features of the abdominal and spinal back muscles in patients with idiopathic scoliosis to those of healthy individuals. The objective was to elucidate the mechanical adaptations in spinal muscles among IS patients.

**Methods:**

This cross-sectional study included 38 patients diagnosed with idiopathic scoliosis and 33 healthy controls. Outcome measures comprised the Cobb angle, spinal curvature, muscle thickness, and muscle elasticity. Ultrasound elastography imaging was employed to assess the thickness and elasticity of the erector spinae, rectus abdominis, external oblique, and transverse abdominis muscles bilaterally at corresponding spinal levels. The objective was to document and compare the ultrasonic imaging characteristics of these muscles in individuals with idiopathic scoliosis and in the normal population.

**Results:**

The study findings indicated that idiopathic scoliosis patients had significantly lower body weight than the control group, with C7-CSVL notably greater in the idiopathic scoliosis group than in healthy individuals. Muscle thickness was substantially reduced on both the concave and convex sides at T6, T10, and L3 levels of the erector spinae, as well as in the rectus abdominis (RA) and transverse abdominis (TrA) muscles, relative to the normal cohort. Additionally, idiopathic scoliosis patients exhibited increased elasticity in the erector spinae muscle on the convex side at T6, while the elasticity of the erector spinae muscle on the concave side at L3 was significantly lower compared to healthy individuals.

**Conclusions:**

This study, utilizing ultrasound elastography imaging technology, unveiled distinct features in individuals with mild idiopathic scoliosis, including decreased muscle thickness in the erector spinae at T6, T10, and L3 levels, as well as heightened elasticity in the thoracic region and reduced elasticity in the lumbar region. The findings presented in this study provide insights for diagnostic strategies in individuals with early-stage scoliosis.

## Introduction

Idiopathic scoliosis (IS) is a condition defined by a three-dimensional deformity of the spine, primarily affecting adolescents [1]. The pathogenesis of IS is intricate, with a significant role attributed to soft tissues, particularly alterations in the function and structure of spinal muscles [2]. Research indicates that individuals with IS demonstrate noteworthy disparities in muscle symmetry on either side of the spine, as well as variations in muscle activation patterns and functionality, which are likely linked to spinal deformity and mechanical imbalance [3, 4].

In recent years, ultrasound imaging techniques have become a widely-utilized tool in the clinical and research fields for non-invasive, real-time, and convenient assessment of muscle structure and function. The introduction of ultrasound elastography technology has facilitated the quantitative evaluation of mechanical characteristics of muscle tissue, including thickness and elasticity. This innovative approach allows for visual analysis of muscle structure and function, offering a fresh research perspective on adaptive changes in muscles among patients with IS. Studies have validated the equivalency of ultrasound imaging in depicting spinal curvature to that of MRI images [5]. Real-time shear-wave elastography (SWE) serves as a non-invasive ultrasound technique that assesses the mechanical properties of muscle tissue by gauging shear modulus value [6]. The relationship between shear modulus and muscle contraction varies; it increases with contraction intensity during isometric contractions and with muscle length during isotonic contractions, thus enabling deductions about muscle mechanical properties. The reliability of SWE technology in evaluating muscles and surrounding tissues relevant to scoliosis has been well-documented [7, 8]. While various researchers have utilized ultrasound imaging to examine the characteristics of the erector spinae and abdominal muscles in IS patients, to the best of our knowledge, no prior study has simultaneously assessed both muscle thickness and elasticity in IS using ultrasound elastography.

This study employed ultrasound elastography imaging technology for the first time to assess the thickness and elastic properties of the abdominal and paraspinal muscles in patients with IS, in comparison with a control group of healthy participants. The primary objective was to uncover the mechanical adaptations in spinal muscles among IS patients. The findings of this study are expected to serve as partial evidence in ultrasound imaging to elucidate the muscle characteristics of idiopathic scoliosis and provide early and non-invasive diagnostic methods for scoliosis.

## Methods

### Research design

This study is a cross-sectional investigation designed to compare the differences in the thickness and elasticity of the back and abdominal muscles between individuals with IS and healthy age-matched controls. The research carried out from September 2023 to March 2025 at the Third People’s Hospital affiliated with Fujian University of Traditional Chinese Medicine. The research protocol has received approval from the ethics committee of our institution (Ethical number: 2023-kl-047) and was registered in the Chinese Clinical Trial Registry (ChiCTR2300074648). All participants have provided informed consent before their involvement in the study.

### Patient selection

#### Inclusion criteria are as follows

IS group: diagnosed with idiopathic scoliosis, aged 18–20, with a Cobb angle of 10° or more, and no history of surgery or other interventions; the level of activity is considered moderate, with regular exercise not exceeding a total of 150 min per week. Control group: healthy adolescents matched in age and gender with the IS group, without spine-related diseases.

#### Exclusion criteria include

Presence of neuromuscular diseases, congenital spine deformities, or systemic diseases; Received systemic rehabilitation or back muscle injection therapy in the past three months; Unable to cooperate in completing the assessment procedures.

### Assessment

This study utilizes ultrasound elastography imaging technology to assess the thickness and elasticity of the back and abdominal muscles in the participants. High-frequency musculoskeletal ultrasound is employed to assess the thickness and elasticity of distinct muscles, encompassing the erector spinae on bilateral sides of the spine at T6, T10, and L3 levels, as well as the rectus abdominis, external oblique, and transverse abdominis. It aims to document and characterize the ultrasound imaging features of individuals with IS in comparison to a healthy control group. The high-frequency musculoskeletal ultrasound device employed is the Siemens-Sequoia Silver, a high-frequency color ultrasound diagnostic equipment manufactured in Germany, operating at frequencies ranging from 5 to 12 MHz. The assessments are carried out by two certified physicians with expertise in ultrasonography, both simultaneously present; one performs the procedure while the other guides the patient’s respiration and monitors their positioning.

#### Examination method

1. *Measurement of Erector Spinae and Abdominal Muscle Thickness* Position the subject in a prone position for the erector spinae muscles and a supine position for the abdominal muscles on a treatment bed with hands placed at the sides to maintain a horizontal spine alignment. Utilize spinous process palpation to identify the sixth thoracic vertebra spinous process (T6), tenth thoracic vertebra spinous process (T10), and third lumbar vertebra spinous process (L3). Mark points approximately 2 cm lateral to the vertebral bodies where the muscles are most prominent. Then, mark the upper, middle, and lower points on both sides and proceed with high-frequency ultrasound scanning at these points. In the supine position, instruct the patient to slightly engage the abdomen to visualize the rectus abdominis (RA) muscles on both sides of the midline. Use a marking pen to guide the ultrasound probe with coupling agent to visualize hypoechoic muscle bundles adjacent to the linea alba. Angle the ultrasound probe downward from ribs 5–12 to locate the external oblique (EO) muscle. Position the ultrasound probe transversely at the designated point to observe the external oblique and transversus abdominis muscle fibers. Place the ultrasound probe with coupling agent on the skin surface, align it with the marked point, capture clear and stable images, and measure muscle thickness by placing cursors on the upper and lower parallel hyperechoic bands, with the vertical distance between them indicating muscle thickness. Take three measurements and calculate the average. Instruct the patient to breathe in and out and capture the muscle image at the end of exhalation. Maintain perpendicular probe position, moderate pressure, and avoid tilting throughout the scan. Ensure image acquisition is consistent among operators.
2. *Measurement of Erector Spinae and Abdominal Muscle Elasticity* In the same positions as above, we employed the Virtual Touch 2D-SWE mode of the Siemens-Sequoia Silver system to measure muscle elasticity, thereby obtaining two-dimensional elastograms and enhancing the visualization of regional comparisons. Adjust the region of interest to 3 mm to cover the erector spinae at T6, T10, L3, rectus abdominis, external oblique, and transversus abdominis completely. Analyze the tissue elasticity map, and the system will automatically calculate muscle tissue elasticity values within the region.
3. C7 to central sacral vertical line (C7-CSVL) distance [9] was the distance between C7 plumbline and the midpoint of the S1 endplate (mm); Iliac to central sacral vertical line (Iliac-CSVL) distance was the the distance between the most superior aspect of the iliac crest and the midpoint of the S1 endplate (mm) on the X-ray image.

### Statistical analysis

All data underwent statistical processing utilizing SPSS 26.0 software. Quantitative data were expressed as mean ± standard deviation (Mean ± SD), and intergroup comparisons were performed through independent sample t-tests. In cases where the data deviated from a normal distribution, either the Mann-Whitney U test or the Wilcoxon signed-rank test was applied. Statistical significance was set at *P* < 0.05. The effect sizes (Cohen’s d) were computed for both groups concurrently, utilizing d values of 0.2, 0.5, and 0.8 to denote small, medium, and large effects, respectively, in evaluating the practical significance of discrepancies.

## Results

Thirty-eight patients with IS and thirty-three normal subjects were selected from a cohort of 200 individuals based on the inclusion criteria. The IS cohort exhibited a lower body weight (52.74 ± 7.25 vs. 56.89 ± 5.37 kg) compared to the control group (*P* < 0.01). However, no statistically significant variances were found between the groups regarding age, height, and BMI (*P* > 0.05). The general characteristics of the subjects are detailed in Table 1.

**Table 1:** Baseline characteristics of study Participants[$\bar {x}\, \pm \,s$, M(P25, P75)]

	IS(*n* = 38)		Control(*n* = 33)		t/Z value	*P* value
Sex Female	28(73.7%)		27(71.1%)		/	/
Male	10(26.3%)		11(28.9%)			
Age (y)^#^	19.46(19.16,19.76)		19.32(19.02,19.62)		−0.787	0.431
Hight (m)^#^	1.63(1.61,1.65)		1.68(1.65,1.72)		−1.939	0.053
Weight (Kg)	**52.74 ± 7.25**		**56.89 ± 5.37**		**−2.885**	**0.005** ^**^
BMI^a^	19.60 ± 2.17		20.67 ± 4.26		−1.305	0.196

T	T-L	L
Segmental Region of Onset for Scoliosis		
39.47% (15/38)	26.32% (10/38)	34.21%(13/38)

^#^Non-parametric tests were employed to analyze data that did not follow a normal distribution.

^a^BMI (Body Mass Index)

^**^Compared to the control group, *P* < 0.01

The results from Table 2 show that the IS group exhibited higher values for Cobb angle [15.38(13.90, 16.86)° vs. 4.89(3.45, 6.34)°], C7-CSVL [4.62(3.55, 5.69) vs. 2.14(1.52, 2.75) mm] compared to the control group (*P* < 0.01), whereas no statistically significant differences were observed for other variables (*P* > 0.05).

**Table 2:** Cobb angle, spinal curvature between group analysis [$\bar {x}\, \pm \,s$, M(P25, P75)]

	IS(*n* = 38)	Control(*n* = 33)	t/Z	*P* valus
**Cobb angle(°)** ^**#**^	**15.38(13.90**,**16.86)**	**4.89(3.45**,**6.34)**	**-6.997**	**0.000** ^***^
Thoracic kyphosis(**°**)^#^	26.37(23.36,29.37)	27.50(24.83,30.17)	-0.729	0.466
Lumbar lordosis(**°**)	45.32 ± 9.83	48.68 ± 9.63	-1.066	0.290
Sacral slope(**°**)^#^	34.78(31.25,38.31)	36.63(33.84,39.41)	-0.589	0.556
**C7-CSVL (mm)** ^**#**^	**4.62(3.55**,**5.69)**	**2.14(1.52**,**2.75)**	**-3.156**	**0.002** ^**^
Rib-CSVL (mm) ^#^	3.37(2.53,4.21)	2.71(1.81,3.61)	-0.941	0.347
Iliac-CSVL (mm)	2.67 ± 1.11	1.62 ± 0.96	0.296	0.768

^#^Non-parametric tests were employed to analyze data that did not follow a normal distribution.

^**^Compared to the control group, *P* < 0.01

^***^Compared to the control group, *P* < 0.001

The results presented in Table 3 indicate that the IS group exhibited decreased muscle thickness compared to the control group for several parameters: T6 ES convex side (0.86(0.76, 0.96) vs. 1.10(0.96, 1.23)), T10 ES concave side (1.15(1.03, 1.27) vs. 1.55(1.38, 1.72)), T10 ES convex side (1.22(1.11, 1.34) vs. 1.65(1.46, 1.84)), L3 concave side (1.72(1.47, 1.97) vs. 2.64(2.41, 2.88)), L3 ES convex side (1.74(1.51, 1.96) vs. 2.45(2.27, 2.64)), RA concave side (0.82 ± 0.21 vs. 1.01 ± 0.27), OA convex side (0.33(0.31, 0.35) vs. 0.40(0.36, 0.43)), and Tra convex side (0.34 ± 0.06 vs. 0.38 ± 0.08) (*P* < 0.05). However, no statistically significant differences were observed for other parameters (*P* > 0.05). Notably, muscle thickness at the concave and convex sides of T10 ES and L3 ES, as well as at the convex side of the OA, demonstrated large effect sizes (Cohen’s d > 0.8).

**Table 3:** Muscle thickness (Ultrasound) between group analysis [$\bar {x}\, \pm \,s$, M(P25, P75)]

Muscles	Concave /Convex	Scoliosis Group(*n* = 38)	Control Group(*n* = 33)	t/Z	*P* valus	Cohen’s d
T6 ES(cm)	Concave Side^#^	0.83(0.74, 0.91)	1.03(0.88, 1.17)	-1.595	0.111	-0.57
	**Convex Side** ^#^	**0.86(0.76**,** 0.96)**	**1.10(0.96**,** 1.23)**	**-2.406**	**0.016** ^*^	**-0.67**
T10 ES(cm)	**Concave Side** ^#^	**1.15(1.03**,** 1.27)**	**1.55(1.38**,** 1.72)**	**-3.408**	**0.001** ^**^	**-0.89**
	**Convex Side** ^#^	**1.22(1.11**,** 1.34)**	**1.65(1.46**,** 1.84)**	**-3.356**	**0.001** ^**^	**-0.92**
L3 ES(cm)	**Concave Side** ^#^	**1.72(1.47**,** 1.97)**	**2.64(2.41**,** 2.88)**	**-4.660**	**0.000** ^***^	**-1.24**
	**Convex Side** ^#^	**1.74(1.51**,** 1.96)**	**2.45(2.27**,** 2.64)**	**-4.058**	**0.000** ^***^	**-1.11**
RA(cm)	**Concave Side**	**0.82 ± 0.21**	**1.01 ± 0.27**	**-3.528**	**0.001** ^**^	**-0.79**
	Convex Side^#^	0.94(0.80, 1.07)	1.03(0.92, 1.13)	-1.725	0.084	-0.24
OA(cm)	Concave Side^#^	0.56(0.18, 0.93)	0.42(0.38, 0.45)	1.857	0.063	-0.43
	**Convex Side** ^#^	**0.33(0.31**,** 0.35)**	**0.40(0.36**,** 0.43)**	**-3.765**	**0.000** ^***^	**-0.86**
TrA(cm)	Concave Side^#^	0.40(0.23, 0.57)	0.55(0.11, 0.99)	-0.318	0.751	-0.15
	**Convex Side**	**0.34 ± 0.06**	**0.38 ± 0.08**	**-2.229**	**0.029** ^*^	**-0.57**

^#^Non-parametric tests were employed to analyze data that did not follow a normal distribution.

^*^Compared to the control group, *P* < 0.05

^**^Compared to the control group, *P* < 0.01

^***^Compared to the control group, *P* < 0.001

The results presented in Table 4 demonstrate that in the IS group, the muscle elasticity of the T6 ES convex side [13.71(11.57, 15.84) vs. 9.02(7.13, 10.91)] was significantly higher than in the control group (*P* < 0.001), whereas the muscle elasticity of the L3 ES concave side [8.70(7.34, 10.05) vs. 10.78(9.30, 12.25)] was notably lower than in the control group (*P* < 0.01), with no statistically significant differences observed for other parameters (*P* > 0.05). A moderate effect size (Cohen’s d > 0.5) was observed in the elasticity on the convex side at T10 ES.

**Table 4:** Muscle elasticity (Ultrasound) between group analysis [M(P25, P75)]

Muscles	Concave /Convex	Scoliosis Group(*n* = 38)	Control Group(*n* = 33)	Z	*P* valus	Cohen’s d
T6 ES (kPa)	Concave Side^#^	13.11(10.49, 15.73)	10.83(8.31, 13.35)	1.928	0.054	0.29
	**Convex Side** ^#^	**13.71(11.57**,** 15.84)**	**9.02(7.13**,** 10.91)**	**3.746**	**0.000** ^***^	**0.76**
T10 ES (kPa)	Concave Side^#^	10.33(7.96, 12.70)	7.69(6.80, 8.59)	0.899	0.369	0.47
	Convex Side^#^	9.48(7.47, 11.49)	7.95(6.73, 9.18)	0.749	0.454	0.30
L3 ES (kPa)	Concave Side^#^	7.54(6.60, 8.47)	9.41(7.86, 10.95)	-1.756	0.079	-0.49
	**Convex Side** ^#^	**8.70(7.34**,** 10.05)**	**10.78(9.30**,** 12.25)**	**-2.297**	**0.022** ^*^	**-0.48**
RA (kPa)	Concave Side^#^	8.04(6.03, 10.05)	7.98(5.78, 10.17)	-0.494	0.622	0.01
	Convex Side^#^	8.82(6.68, 10.96)	7.08(5.63, 8.54)	-1.055	0.292	0.31
OA (kPa)	Concave Side^#^	12.52(10.57, 14.47)	11.13(8.66, 13.60)	1.522	0.128	0.21
	Convex Side^#^	11.52(9.62, 13.42)	12.16(10.34, 13.98)	-0.068	0.946	-0.11
TrA (kPa)	Concave Side^#^	7.44(6.18, 8.69)	7.50(5.28, 9.72)	-1.138	0.255	-0.01
	Convex Side^#^	7.14(5.60, 8.39)	6.19(4.89,7.49)	1.575	0.115	0.25

^#^Non-parametric tests were employed to analyze data that did not follow a normal distribution.

^*^Compared to the control group, *P* < 0.05

^***^Compared to the control group, *P* < 0.001

## Discussion

This study employed ultrasound imaging technology to systematically assess the variations in spinal and abdominal muscle thickness and elasticity between individuals with mild IS and healthy controls. It was evident that the weight of IS patients was notably lower compared to the control group, aligning with findings from previous studies. A comprehensive review revealed that individuals with scoliosis commonly exhibit tall stature and low body mass index (BMI) [10]. A study conducted in South Korea demonstrated a strong correlation between low body weight and the susceptibility to scoliosis, implying that maintaining a proper weight in elementary school students could serve as an effective strategy in preventing and mitigating the risks associated with scoliosis [11, 12]. Yang et al. [13] highlighted that IS patients generally have reduced muscle mass, particularly in the spinal area, indicating that delayed muscle development might contribute to the pathogenesis of IS. The discrepancy in weight observed in our study could be linked to the overall decline in muscle thickness, chiefly stemming from insufficient development of paraspinal and core muscle groups.

In this research, the thickness of the spinal muscles at T6, T10, and L3 in patients with IS, along with the RA and TrA muscles, showed significant reduction on both sides, concave and convex, compared to those of healthy individuals. Upon examining patients with IS, it becomes evident that the primary regions of onset are predominantly localized in the thoracolumbar segment. The findings of this study highlight that the variations in muscle thickness primarily demonstrate a correlation between the thoracolumbar and onset segments. Supporting this, Zhou et al. [14] and Zapata [15] identified a decline in muscle volume of paraspinal muscles and an increase in muscle fat infiltration in IS patients using three-dimensional reconstruction. Morphological and histological studies have indicated that the diminished muscle strength in individuals with IS can be attributed to increased fibrosis, fat degeneration, and a rise in type II muscle fibers [16]. It has been widely documented through various studies that muscle imbalance is the primary cause of IS [17, 18], supporting a hypothesis of muscle equilibrium, wherein the spinal muscles strive to align the spine in a neutral position [17]. Consequently, the utilization of ultrasound examination in prospective clinical scenarios holds promise for identifying these specific alterations and aiding in the diagnosis of scoliosis, providing a novel diagnostic tool.

Furthermore, the decreased thickness of RA and TrA indicates the presence of core stability deficits in IS patients. Borna et al. [19] assessed the thickness, symmetry, and activation of the external oblique (EO), internal oblique (IO), and TrA muscles in AIS patients, revealing a notable decrease in the EO and TrA muscle thickness compared to that of the healthy control group. However, it has also been reported that the muscle thickness changes measured is correlated well with electromyographic activity and that it is highly correlated with the TrA muscle thickness calculated by magnetic resonance imaging [20, 21]. Therefore, the muscle size can provide an indirect measure of the force-generating capacity as shown for a variety of muscles [22].This finding aligns with our discovery of reduced muscle thickness, suggesting potential early involvement of the core muscle group, which could lead to compromised trunk control and exacerbation of spinal rotation and deviation. Notably, large effect sizes (Cohen’s d >0.8) in muscle thickness at the concave and convex sides of T10 and L3, as well as at the convex side of the OA, suggest pronounced asymmetries in paraspinal morphology.

In the IS group, the C7-CSVL distances were notably greater compared to those in healthy controls. These results signify a pronounced coronal plane misalignment, implying an imbalance in trunk posture of IS patients [9]. The increased C7-CSVL measurement indicates a displacement of the head and upper trunk away from the central sacral line, aligning with the well-known three-dimensional rotational deformity characteristic of IS. These variations underscore the role of spinal alignment in IS, highlighting the need for comprehensive postural evaluation and targeted intervention strategies. Moreover, such global misalignment may be associated with muscle asymmetries, which could in turn contribute to postural instability and inefficient movement patterns in this population.

Prior research [23] has indicated that a common characteristic of IS is a decrease in thoracic kyphosis; however, no such distinction was observed in this study. This discrepancy could potentially be attributed to the inclusion of patients with milder spinal curvature and a limited sample size. To validate these findings, future studies should consider augmenting the sample size and utilizing various imaging modalities.

Ultrasound elastography assessments in the supine position revealed intriguing findings in patients with IS. Specifically, the elasticity of the spinal muscles on the convex side of T6 was higher in IS patients compared to the control group, while the elasticity of the paravertebral muscles on the concave side of L3 was considerably lower in IS patients than in normal individuals. These site-specific alterations in elasticity could signify varying muscle adaptation responses across distinct spinal segments. The elevated elasticity observed on the convex side of T6 may indicate compensatory muscle tension or spasms in that region, supporting the concept of stress asymmetry between the concave and convex sides [24]. Conversely, the reduced elasticity detected on the concave side of L3 may suggest a combination of muscle atrophy and diminished elasticity, implying potential chronic disuse or alteration in movement patterns. In addition, the moderate effect size observed in muscle elasticity at the convex side of the erector spinae at T10 (Cohen’s d > 0.5) indicates that structural alterations are accompanied by functional differences in tissue properties. While significant differences in muscle thickness were noted in specific regions, no notable discrepancies in muscle elasticity were identified between the IS and control groups. This could be linked to the relatively mild scoliosis severity (mean Cobb angle 15°) within the study sample, possibly indicating underdeveloped muscle alterations. Moreover, variations in spinal curvature patterns and soft tissue composition among individuals might have obscured subtle group distinctions. Constraints in measurement, such as limited ultrasound penetration in deep muscles or the impact of non-functional testing posture (supine position), may have also played a role. Even in mild deformities, early muscle adaptations can be observed. To better characterize these changes in IS patients, future studies could combine surface electromyography (sEMG) with dynamic ultrasound assessments to provide more comprehensive insights.

This study indicates that significant structural changes in muscle thickness are present in patients with mild IS even with a Cobb angle of approximately 15°. As a result, rehabilitation programs should prioritize the strengthening of muscle groups including the erector spinae [25], rectus abdominis, and transverse abdominis [26]. Subsequently, future assessment tools could potentially incorporate the muscle thickness and elasticity of the erector spinae and abdominal muscles in IS patients as clinical predictive indicators. Ultrasound elastography demonstrates potential as a non-invasive monitoring tool, but its clinical role requires further validation.

## Limitation

This study is designed as a cross-sectional analysis, which does not allow for the establishment of causal relationships. The relatively small sample size underscores the importance of conducting future multicenter studies with larger sample sizes. Muscle functionality, including factors like endurance and activation patterns, was not assessed. Therefore, it is advisable to incorporate electromyography or dynamic ultrasound tests in forthcoming investigations. For future research, employing a longitudinal study design to monitor the progression of muscle adaptation and its predictive significance in the evolution of spinal curvature. Undertaking extensive multicenter cohort studies that encompass younger adolescents and individuals with diverse scoliosis severities. Integrating electromyography, muscle strength assessments, and endurance measurements to delve deeper into the interplay between structure and function.

## Conclusion

This study employed ultrasound imaging technology to uncover that individuals with mild IS demonstrate features including decreased muscle thickness in the erector spinae at T6, T10, L3, and in the rectus abdominis and transverse abdominis, heightened elasticity on the thoracic segment’s one side, and reduced elasticity on the lumbar segment’s one side. However, no significant changes were observed in many muscles, which may be attributed to the relatively mild degree of spinal curvature (mean Cobb angle ≈ 15°). These findings suggest that early-stage scoliosis is characterized by selective rather than generalized muscle alterations. Importantly, the results highlight the potential value of ultrasound imaging as a sensitive and non-invasive tool for the early detection and monitoring of muscle adaptations in individuals with idiopathic scoliosis.

## Data Availability

All data generated or analyzed during this study are included in this article.
